# Mesenchymal stem cells stimulate intestinal stem cells to repair radiation-induced intestinal injury

**DOI:** 10.1038/cddis.2016.276

**Published:** 2016-09-29

**Authors:** Wei Gong, Mengzheng Guo, Zhibo Han, Yan Wang, Ping Yang, Chang Xu, Qin Wang, Liqing Du, Qian Li, Hui Zhao, Feiyue Fan, Qiang Liu

**Affiliations:** 1 Tianjin Key Laboratory of Radiation Medicine and Molecular Nuclear Medicine, Department of Radiobiology, Institute of Radiation Medicine of Chinese Academy of Medical Science and Peking Union Medical College, Tianjin, China; 2 Institute of Hematology and Blood Disease Hospital, Chinese Academy of Medical Sciences and Peking Union of Medical College, Tianjin, China; 3 Tianjin Institute of Medical and Pharmaceutical Sciences, Tianjin, China; 4 Tianjin Key Laboratory of Food and Biotechnology, School of Biotechnology and Food Science, Tianjin University of Commerce, Tianjin, China; 5 Department of Hematology and Translation Medicine Centre, North China University of Science and Technology Affiliated Hospital, Tangshan, China; 6 Institute of Laboratory Animal Sciences of Chinese Academy of Medical Science and Peking Union Medical College, Beijing, China

## Abstract

The loss of stem cells residing in the base of the intestinal crypt has a key role in radiation-induced intestinal injury. In particular, Lgr5^+^ intestinal stem cells (ISCs) are indispensable for intestinal regeneration following exposure to radiation. Mesenchymal stem cells (MSCs) have previously been shown to improve intestinal epithelial repair in a mouse model of radiation injury, and, therefore, it was hypothesized that this protective effect is related to Lgr5^+^ ISCs. In this study, it was found that, following exposure to radiation, transplantation of MSCs improved the survival of the mice, ameliorated intestinal injury and increased the number of regenerating crypts. Furthermore, there was a significant increase in Lgr5^+^ ISCs and their daughter cells, including Ki67^+^ transient amplifying cells, Vil1^+^ enterocytes and lysozyme^+^ Paneth cells, in response to treatment with MSCs. Crypts isolated from mice treated with MSCs formed a higher number of and larger enteroids than those from the PBS group. MSC transplantation also reduced the number of apoptotic cells within the small intestine at 6 h post-radiation. Interestingly, Wnt3a and active *β*-catenin protein levels were increased in the small intestines of MSC-treated mice. In addition, intravenous delivery of recombinant mouse Wnt3a after radiation reduced damage in the small intestine and was radioprotective, although not to the same degree as MSC treatment. Our results show that MSCs support the growth of endogenous Lgr5^+^ ISCs, thus promoting repair of the small intestine following exposure to radiation. The molecular mechanism of action mediating this was found to be related to increased activation of the Wnt/*β*-catenin signaling pathway.

The epithelium of the small intestine contains crypts and villi. Intestinal stem cells (ISCs) reside in the base of the crypts and are responsible for maintaining intestinal epithelial homeostasis and regeneration following injury.^1, 2^ Recent studies have identified two populations of stem cells in the small intestine of mice called Lgr5^+^ and Bmi1^+^ ISCs.^3, 4, 5, 6, 7, 8, 9, 10, 11^ Lgr5^+^ ISCs, also known as crypt base columnar cells (CBCs), are interspersed among the Paneth cells and are active rapidly cycling stem cells.^12^ A single Lgr5^+^ ISC can grow to form ‘enteroids' *in vitro* that develop into all the differentiated cell types found in the intestinal crypt.^13^ Conversely, Bmi1^+^ cells are a population of ISCs located at position +4 relative to the base of the crypt, and are quiescent, slowly cycling stem cells.^14^ The loss of ISCs has a critical role in radiation-induced intestinal injury (RIII).^15, 16, 17, 18^ Apoptosis of stem cells because of exposure to radiation prevents normal re-epithelialization of the intestines. Therefore, enhancing the survival of ISCs following radiation is a potential effective treatment for RIII.

Mesenchymal stem cells (MSCs) possess significant potential as a therapeutic for tissue damage because of their ability to regulate inflammation, inhibit apoptosis, promote angiogenesis, and support the growth and differentiation of local stem and progenitor cells.^19, 20^ However, the mechanisms by which MSCs mediate these beneficial effects remain unclear, although it has been suggested that MSCs may actively secrete a broad range of bioactive molecules with immunomodulatory (PGE2, IDO, NO, HLA-G5, TSG-6, IL-6, IL-10 and IL-1RA), mitogenic (TGF*α*/*β*, HGF, IGF-1, bFGF and EGF), angiogenic (VEGF and TGF-*β*1) and/or anti-apoptotic (STC-1 and SFRP2) properties that function to modulate the regenerative environment at the site of injury.^21^ Upon re-establishment of the microenvironment following damage, the surviving endogenous stem and progenitor cells can then regenerate the injured tissue completely.

Our previous study, as well as other published studies, has found that systemic administration of MSCs improves intestinal epithelial repair in an animal model of radiation injury.^22, 23, 24, 25^ Following MSC treatment, radiation-induced lesions in mice were significantly smaller than those in the control group. However, the mechanism behind this protective effect is not fully understood. Lgr5^+^ ISCs have been previously shown to be indispensable for radiation-induced intestinal regeneration.^26^ Therefore, in this study, we tested whether the therapeutic effects of MSCs in response to RIII are related to the Lgr5^+^ population of resident ISCs.

## Results

### MSCs improve the survival of mice after ABI

In order to limit damage to other organs and tissues, such as bone marrow, abdominal irradiation (ABI) was used instead of total body irradiation (TBI) to cause radiation-induced intestinal damage. As exposure of the gastrointestinal tract of mice to doses of 12 Gy or lower is considered nonlethal,^16, 27^ we administered escalating doses of 12, 14 and 16 Gy of ABI to C57BL/6 mice to evaluate the radioprotective effects of MSCs.

**Figure 1:**
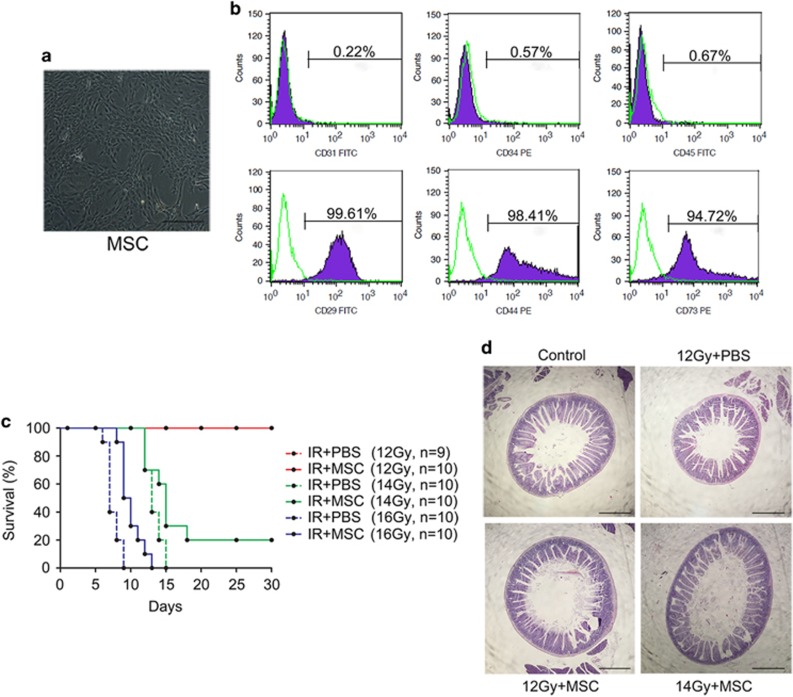
MSCs improve the survival of mice exposed to 14 Gy ABI. (**a**) Spindle-like morphology of BM-MSCs at passage 3. Bars, 400 *μ*m. (**b**) Flow cytometry demonstrating BM-MSCs were negative for CD31, CD34 and CD45, and positive for CD29, CD44 and CD73. (**c**) Kaplan–Meier survival curve of C57BL/6 mice treated with PBS or MSCs after 12, 14 or 16 Gy ABI. Of the MSC-treated mice, 20% survived to 30 days post-14 Gy radiation. The number of animals in each treatment group is shown in parentheses. (**d**) Representative H&E-stained small intestine sections from mice surviving to day 30. Bars, 1000 *μ*m

First, MSCs isolated from the bone marrow of C57BL/6 mice were characterized. MSCs that were cultured *in vitro* had a spindle-like morphology and were adherent to tissue culture plastic (Figure 1a). Flow cytometry revealed that these cells were negative for CD31, CD34 and CD45, and positive for CD29, CD44 and CD73 (Figure 1b).

Exposure to 12 Gy of radiation caused no mouse mortality, although there was a decrease in body weight within 30 days post-radiation. When exposed to 14 Gy of radiation, 20% of the mice treated with MSCs survived, while none of the PBS-treated mice exposed to this amount of radiation survived to 30 days. At 16 Gy, all the mice died by 30 days; however, the MSC-treated mice lived longer compared with the PBS-treated group (Figure 1c).

On day 30 post-radiation, all of the surviving mice, which were from 12 Gy PBS-treated group, 12 Gy MSC-treated group, and 14 Gy MSC-treated group, were killed, and the small intestines were harvested for evaluation by histology. H&E-staining of sections of the small intestine revealed that the mucosa was well preserved after radiation, and was indistinguishable from non-irradiated intestinal tissue (Figure 1d).

### MSCs promote repair after radiation-induced damage to the small intestines

**Figure 2:**
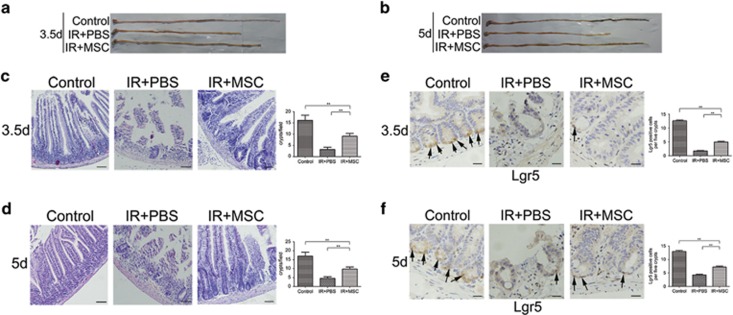
MSC administration results in less damage to the small intestine and an increased number of Lgr5^+^ ISCs in mice following 14 Gy ABI. Small intestines from PBS-treated mice were shorter than those from MSC-treated mice at 3.5 (**a**) and 5 (**b**) days after radiation. In H&E-stained sections, crypt-villus architecture of MSC-treated mice was well preserved, and the number of crypts had significantly increased by days 3.5 (**c**) and 5 (**d**) post-radiation compared with PBS-treated mice. Crypts in a single field of view were quantified. Bars, 100 *μ*m. MSC administration resulted in an increased number of Lgr5-positive cells (indicated with arrows) at 3.5 (**e**) and 5 (**f**) days post-radiation. Lgr5-positive cells were quantified in five crypts per mouse. Bars, 50 *μ*m. Results are shown as mean±S.D. ^**^P<0.01

The amount of damage to the small intestines of mice following 14 Gy ABI with or without subsequent MSC transplantation was evaluated at 3.5 and 5 days post-radiation. As shown in Figure 2a, the average length of the small intestines from PBS-treated mice was shorter than that of MSC-treated mice at 3.5 days after radiation. In addition, the crypt-villus architecture in the small intestines of mice in the PBS group was significantly interrupted. Specifically, only a small number of intestinal crypts remained, and the villi were stunted. By contrast, the crypt-villus architecture in mice treated with MSCs was well preserved, although there were fewer crypts than in the control group (Figure 2c). Five days post-radiation, the length of the small intestines from PBS-treated mice remained significantly shorter, whereas small intestines from the MSC-treated mice were as long as those from the non-irradiated control group mice (Figure 2b). Furthermore, the mucosa of PBS-treated mice failed to recover, whereas there was partial repair observed in the MSC-treated mice (Figure 2d).

Lgr5^+^ ISCs have been shown to be indispensable for intestinal regeneration following damage by radiation;^26^ therefore, the population of Lgr5^+^ ISCs was evaluated. It was found that the number of Lgr5^+^ ISCs in PBS-treated mice was significantly decreased at 3.5 (Figure 2e) and 5 days after radiation compared with the control (Figure 2f). By contrast, the Lgr5^+^ ISC population had partially recovered in the small intestines of MSC-treated mice. In addition, the average number of Lgr5^+^ ISCs in the MSC-treated mice was significantly higher than in the PBS group at both 3.5 and 5 days following radiation (Figures 2e and f).

**Figure 3:**
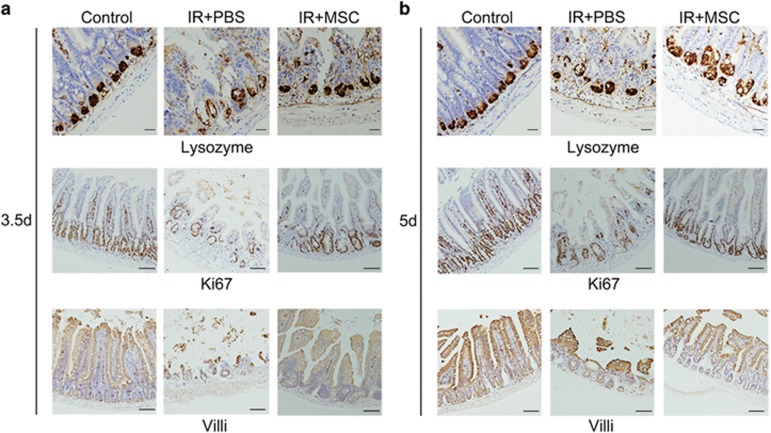
MSC administration resulted in an increase in Lgr5^+^ ISC progeny. MSC transplantation resulted in an increase in Ki67^+^ transiently replicating cells, Vil1^+^ enterocytes and lysozyme^+^ Paneth cells at 3.5 (**a**) and 5 (**b**) days post-radiation. Bars, 50 and 100 *μ*m

The intestinal epithelium has the capacity for substantial regeneration post-injury. ISC divide and produce transiently amplifying (TA) cells, which migrate either upward to the villus to differentiate into functional cells, such as enterocytes, or downward into the base of the crypts to give rise to Paneth cells. At 3.5 days after radiation, the numbers of Ki67^+^ TA cells, Vil1^+^ enterocytes and lysozyme^+^ Paneth cells were markedly lower than the non-irradiated control group, which is consistent with the marked loss of Lgr5^+^ ISCs in the PBS treatment group (Figure 3a). The phenotype was similar at 5 days after radiation (Figure 3b). By contrast, MSC-treated mice had an increase in the numbers of Ki67^+^ TA cells, Vil1^+^ enterocytes and lysozyme^+^ Paneth cells at both 3.5 and 5 days after radiation (Figures 3a and b), correlating with the relative increase in Lgr5^+^ ISCs noted in the MSC treatment group.

### MSCs improve the growth of intestinal crypts *in vitro*

**Figure 4:**
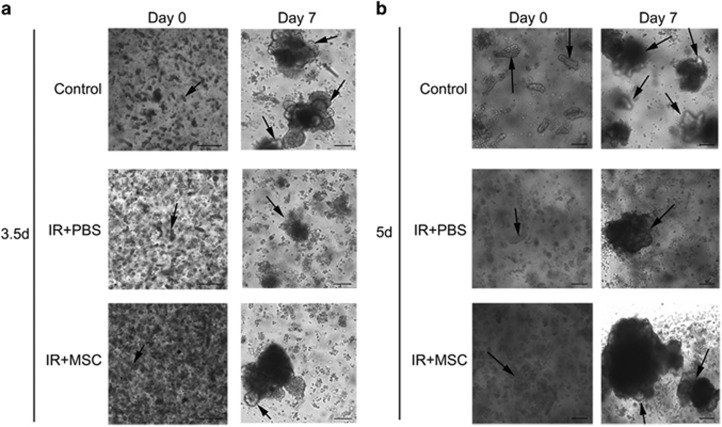
MSC treatment *in vivo* improves the growth of intestinal crypts *ex vivo*. Crypts were obtained from each cohort of mice at 3.5 (**a**) and 5 (**b**) days post-radiation and cultured *in vitro* for 7 days. The intestinal crypts isolated from the MSC-group formed more and larger enteroids (highlighted with arrows) relative to the PBS-treated group. Bars, 400 and 1000 *μ*m

Isolated intestinal crypts or Lgr5^+^ ISCs can be grown *in vitro* to form ‘enteroids' that comprises all the differentiated intestinal cell types found in the intestinal crypt.^13, 28, 29, 30^ This primary cell culture model can be used to simulate the physiology of the intestinal epithelium. Based on the method published by Sato *et al.*, the intestinal crypts of mice that had been exposed to 14 Gy of ABI were isolated and cultured *in vitro*. When collected 3.5 days after radiation, the total number of crypts isolated from PBS-treated mice was significantly lower than from the MSC-treated mice. When the same number of crypts was cultured from each treatment cohort *in vitro*, the intestinal crypts isolated from the MSC treatment group formed more and larger enteroids than crypts from the PBS group (Figure 4a). When collecting crypts to form enteroids at 5 days after radiation, a similar result was observed (Figure 4b).

### MSCs decrease the rate of apoptosis in the small intestine

**Figure 5:**
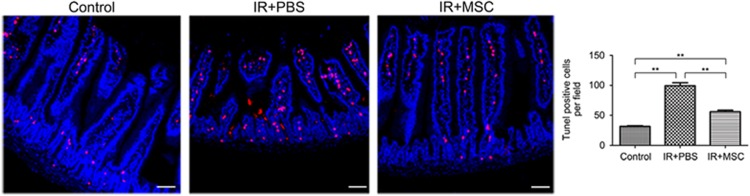
MSCs decrease the rate of apoptosis in the small intestine. At 6 h post-radiation, the frequency of apoptosis in the small intestines was measured using TUNEL staining. TUNEL-positive cells (red) in a single field of view were quantified. Results are shown as mean±S.D. ^**^P<0.01. Bars, 100 *μ*m

Basal levels of apoptosis are always present, even in normal healthy tissue. This type of apoptosis is known as spontaneous apoptosis and it occurs in the small intestine independently of the p53 signaling pathway. By contrast, radiation-induced apoptosis is p53 dependent, and its frequency typically peaks in the small intestine at 6 h after radiation in mice.^15^ Terminal deoxynucleotidyl transferase dUTP nick end labeling (TUNEL) staining of intestinal tissue from mice 6 h after radiation revealed an abundant population of apoptotic cells in the crypt and villi. Importantly, MSC transplantation significantly decreased the size of this population compared with the PBS group (Figure 5).

### MSCs activate the Wnt/*β*-catenin signaling pathway

**Figure 6:**
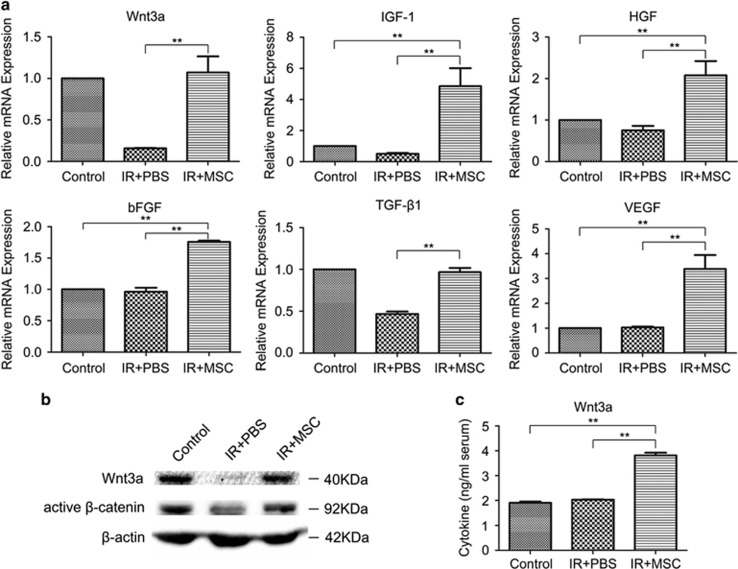
MSCs activate Wnt/*β*-catenin signaling in the small intestines of ABI mice. After 14 Gy ABI, MSC transplantation resulted in increased expression of the growth factors Wnt3a, IGF-1, HGF, bFGF, TGF-*β*1 and VEGF (**a**), and protein levels of Wnt3a and active *β*-catenin at 3.5 days post-irradiation (**b**) in the small intestine, and circulating Wnt3a serum levels at 2 days post-irradiation (**c**). Results are shown as mean±S.D. ^**^P<0.01

**Figure 7:**
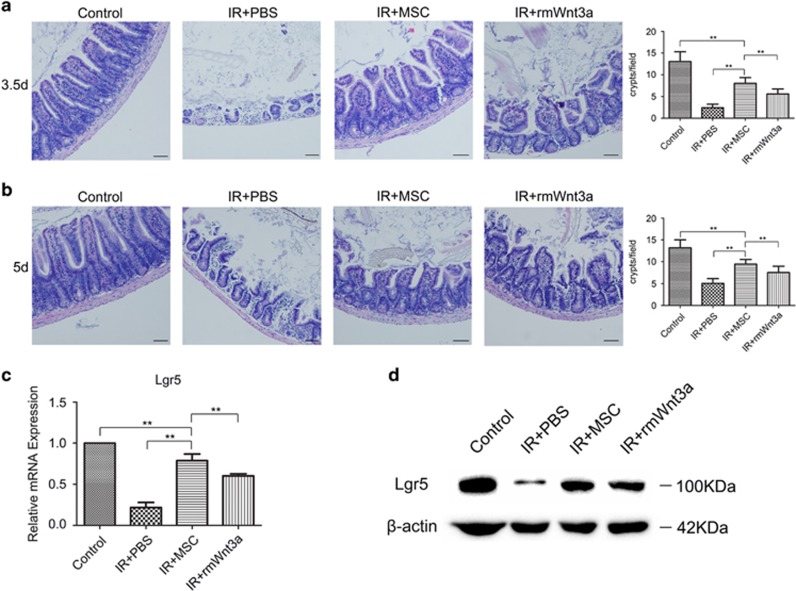
The radioprotective effect of MSCs is partially recapitulated by rmWnt3a. After 14 Gy ABI, intravenous delivery of rmWnt3a alone was enough to alleviate intestinal injury and increase the number of regenerating crypts at 3.5 and 5 (**a**) days post-irradiation. Crypts in a single field of view were quantified. Bars, 100 *μ*m. rmWnt3a treatment was also found to simultaneously increase Lgr5 mRNA (**b**) and protein expression (**c**) in the small intestines of ABI mice at 3.5 days post-irradiation. Results are shown as mean±S.D. ^**^P<0.01

Next, the potential molecular mechanisms involved in the beneficial effects of MSC transplant were investigated. Growth factors have been found to protect against RIII.^31, 32^ Thus, the mRNA expression of a number of growth factors was quantified in the small intestine post-ABI and MSC treatment. The expression of Wnt3a, IGF-1, HGF, bFGF, TGF*β*1 and VEGF mRNA was significantly higher (*P*< 0.01) at day 3.5 in the MSC-treated group compared with the PBS group (Figure 6a). Previous studies have shown that activation of Wnt signaling may be required to induce intestinal regeneration.^33, 34, 35^ Therefore, we measured Wnt3a and active *β*-catenin protein levels in the small intestine following MSC administration, and found a significant increase in both of these proteins compared with the PBS group (Figure 6b). The enhancement of active *β*-catenin levels in the small intestine suggests that the Wnt pathway is activated by MSCs. Furthermore, the Wnt3a protein levels circulating in the serum following MSC treatment were also increased 2 days post-radiation (Figure 6c). Overall, these data suggest a role for Wnt3a in mediating the therapeutic benefits of MSCs. To determine if Wnt3a can substitute for MSCs in the RIII model, recombinant mouse Wnt3a (rmWnt3a) was administered via tail vein injection following exposure to radiation. The amount of intestinal damage from 14 Gy ABI was reduced in the rmWnt3a-treated mice, and rmWnt3a was radioprotective, although only partially protective compared with MSC treatment (Figures 7a and c).

## Discussion

The intestine is one of the most radiosensitive organs in the body. Exposure to high doses of radiation, for example, after a nuclear accident, can cause severe intestinal damage and high rates of mortality. Similarly, clinical use of radiation used to treat tumors in the abdominal and pelvic cavity may also lead to acute and/or chronic intestinal injury. The intestinal side effects of radiation limit the effective radiation dosage that can be applied to eradicate tumors and reduce patient quality of life. Currently, there are no effective treatment strategies available to prevent and/or reduce RIII.

In this study, we observed that MSC transplantation improved the survival of lethally irradiated mice, and promoted the repair of RIII. This protective effect was related to the population of Lgr5^+^ ISCs. Specifically, Lgr5^+^ ISCs, while not required for normal intestinal homeostasis, are indispensable for radiation-induced intestinal regeneration.^36^ After radiation, treatment with MSCs resulted in an increased number of Lgr5^+^ ISCs compared with PBS treatment. Consistent with this, MSC transplantation also increased the number of regenerating intestinal crypts, which is an indicator of stem cell survival and predictive of whether an animal will eventually die from intestinal injury.^27^ Exposure to 14 Gy ABI significantly decreased the number of regenerating crypts, but subsequent MSC transplantation resulted in a comparative increase in crypt formation. In addition, upon culturing intestinal crypts from the different experimental cohorts of mice *in vitro*, the crypts isolated from MSC-treated mice resulted in larger and more ‘enteroids' relative to the PBS treatment group. Furthermore, examination of the downstream progeny of Lgr5^+^ ISCs, such as Ki67^+^ TA cells, Vil1^+^ enterocytes and lysozyme^+^ Paneth cells, revealed that MSC transplantation increased the populations of these types of cells compared with the PBS group. In addition, apoptosis in the small intestines was assessed 6 h post-radiation, and fewer apoptotic cells were found in the small intestines of MSC transplanted irradiated mice than PBS treated.

The molecular mechanisms behind the beneficial effects of the MSCs included amplified activation of the Wnt/*β*-catenin signaling pathway in the small intestines of ABI mice. Wnt/*β*-catenin signaling pathway is crucial for the proliferation and maintenance of ISCs.^33, 34, 35^ Compared with PBS treatment, MSC injection resulted in an increase in both Wnt3a and active *β*-catenin in the small intestines. Furthermore, intravenous delivery of rmWnt3a alone following radiation also alleviated intestinal injury and increased the number of regenerating crypts. However, it is interesting to note that rmWnt3a alone, while protective, only partially recapitulated the effect of MSCs, suggesting that additional soluble factors or combined effects may have a role. Chen *et al.*^24^ showed that IGF-1 has a critical role in recovery from RIII. In our study, we also observed a significant increase in the expression levels of IGF-1 mRNA in the small intestines of ABI mice following MSC treatment. As more cytokines are identified that have a role in remediating intestinal injury, eventually a combination of several factors may be used therapeutically to achieve the benefits of MSC treatment in RIII. These cell-free therapeutics can overcome some limitations of MSC-based therapy.

Although ISCs have a key role in intestinal epithelial regeneration following injury, which population(s) of ISCs that repopulate the intestine following RIII are still unknown. A very recent study has unveiled that Lgr5^+^ ISCs are indispensable for intestinal regeneration following radiation.^26^ In this study, we observed and confirmed that MSCs stimulate endogenous Lgr5^+^ ISC-mediated small intestinal epithelial regeneration in mice irradiated with 14 Gy ABI.

RIII occurs in a dose-dependent manner. A low-dose of radiation (≤1 Gy) induces apoptosis of highly radiosensitive ISCs, but does not lead to intestinal injury. Exposure to a range of 8–14 Gy increases Lgr5^+^ ISC death as the dosage increases; however, the surviving Lgr5^+^ ISCs are still sufficient to support complete intestinal recovery. At 15 Gy, there is more widespread apoptosis of Lgr5^+^ ISCs, resulting in a failure to restore viability of the small intestines.^27^ Fourteen Gy may be the maximum dose to allow for full recovery of the intestines. In our study, 14 Gy eventually caused death in all mice that had been subsequently treated with PBS alone, whereas 20% of mice treated with MSCs survived.

In conclusion, this study demonstrates that MSC transplantation enhances intestinal repair and improves the survival of mice after RIII. We found that MSCs decrease the frequency of apoptosis in the small intestines and support the growth of endogenous Lgr5^+^ ISCs for organ and tissue repair. A molecular mechanism by which the benefits of MSCs are mediated is through amplified activation of the Wnt/*β*-catenin signaling pathway in the small intestines. It is important to conduct further studies on MSCs to further evaluate their potential as a therapeutic agent for radiation enteropathy.

## Materials and Methods

### Mouse model of RIII

Male C57BL/6 mice, aged 6–8 weeks and weighing 23–24 g, were purchased from Vital River Laboratory Animal Technology Co. Ltd (Beijing, China). All mice were housed in a temperature-controlled, specific-pathogen free environment with a 12-h light/dark cycle, and fed standard chow and water. ABI was performed on mice using a Cr^137^*γ*-ray irradiator (Atomic Energy of Canada, Chalk River, Ontario, Canada), where lead shielding was used to protect other parts of the body from irradiation. Mice were exposed to 12, 14 and 16 Gy at 1 Gy/min at room temperature, and 14 Gy was selected as the optimal irradiation dose for future experiments. All experimental procedures and protocols were conducted according to the guidelines of our local animal care and use committee.

### Cell culture

Bone marrow MSCs were isolated from C57Bl/6 mice and expanded using a MesenCult Proliferation Kit (StemCell Technologies, Vancouver, BC, Canada) according to the manufacturer's instructions. Briefly, bone marrow cells were collected from the femur and tibia, and plated in a T25 flask (Corning, NY, USA) in 10 ml complete medium. Once confluent, the adherent cells (passage 0) were detached with 0.25% trypsin (Gibco, Grand island, NY, USA) and passaged by splitting 1 : 3 by volume. Flow cytometry was used to assess cell surface marker expression, such as CD29, CD31, CD34, CD44, CD45 and CD73 (BD Biosciences, Franklin Lakes, NJ, USA), at passage 3.

### *In vivo* treatment with MSCs and recombinant mouse Wnt3a

Within 2 h after irradiation, the mice were injected with either 1 million MSCs (passage 5), 400 ng of recombinant mouse Wnt3a (R&D Systems, Minneapolis, MN, USA), or PBS via tail vein injections in a total volume of 200 *μ*l.

### *In vitro* culture of intestinal crypts

The method by which the mouse intestinal crypts were isolated and cultured *in vitro* was modified from Mahe *et al.*^19^ Briefly, mouse jejunums (∼6 cm) were harvested, sliced and opened longitudinally, washed with ice-cold PBS, cut into small pieces and then incubated in ice-cold PBS containing 2 mM EDTA for 30 min. After rinsing twice with ice-cold PBS, the fragments were resuspended in ice-cold PBS and passed through a 70-mm filter. A total of 500 crypts were resuspended in 20 *μ*l of gut media (advanced DMEM/F12 (Gibco), 50 ng/ml EGF (Sigma-Aldrich, St. Louis, MO, USA), 100 ng/ml Noggin (R&D Systems), 1 *μ*g/ml R-spondin 1 (R&D Systems), 10 mM HEPES (Gibco), 1% penicillin–streptomycin–glutamine (Gibco), 1% N_2_ supplement (Invitrogen, Carlsbad, CA, USA) and 2% B27 supplement (Invitrogen)), mixed with 50 *μ*l Matrigel (BD Bioscience) and then plated in one well of a pre-heated 24-well plate (Corning). This plate was placed in a 37 °C incubator for 30 min, and then 500 *μ*l of gut media was added. The gut media were replaced with fresh media every 4 days.

### RT-PCR

Total RNA was extracted from the small intestines using TRIzol Reagent (Invitrogen). A 10 *μ*g aliquot of each RNA sample was reverse transcribed into cDNA using oligo-dT random primers and reverse transcriptase (Takara, Dalian, China), and RT-PCR was performed using SYBR Premix Ex TaqTM II (Takara). Primers used were as follows:

Wnt3a: 5′-TTCTTACTTGAGGGCGGAGA-3′ (forward) and 5′-ACCCGTATCCCAGACAGGA-3′ (reverse); IGF-1: 5′-CAACTCCCAGCTGTGCAATT-3′ (forward) and 5′-GCCGAGGTGAACACAAAACT-3′ (reverse); HGF: 5′-CAGGACCATGTGAGGGAGAT-3′ (forward) and 5′-TACCAGGACGATTTGGGATG-3′ (reverse); bFGF: 5′-AAGGGAGTGTGTGCCAACC-3′ (forward) and 5′-GCCCAGTTCGTTTCAGTGC-3′ (reverse); VEGF: 5′-CCCTTCGTCCTCTCCTTACC-3′ (forward) and 5′-AAGCCACTCACACACACAGC-3′ (reverse); TGF-*β*1: 5′-ATTCCTGGCGTTACCTTGG-3′ (forward) and 5′-AGCCCTGTATTCCGTCTCCT-3′ (reverse); Lgr5: 5′-CGGAGGAAGCGCTACAGAAT-3′ (forward) and 5′-CTGGGTGGCACGTAGCTGAT-3′ (reverse); and GAPDH: 5′-GGTGGGTGGTCCAAGGTTTC-3′ (forward) and 5′-TGGTTTGACAATGAATACGGCTAC-3′ (reverse).

### Western blots

Protein was extracted from small intestines incubated in ice-cold lysis buffer (Solarbio Science and Technology, Beijing, China). Protein concentrations were quantified using a BCA protein kit (Beyotime, Shanghai, China). Samples containing equal amounts of protein (20 *μ*g) were mixed with loading buffer containing 5% 2-mercaptoethanol, heated for 10 min at 95 °C, and loaded onto a 10% SDS-PAGE gel. After electrophoresing the proteins along the gel, the proteins were then transferred to polyvinylidene difluoride membranes. These membranes were blocked with 5% milk and 0.1% Tween 20 in Tris-buffered saline, and then incubated overnight at 4 °C with primary antibody, that is, anti-Wnt3a (Abcam, Cambridge, MA, USA), anti-activated *β*-catenin (Millipore, Darmstadt, Germany), anti-Lgr5 (Abcam) or anti-*β*-actin (Beijing ComWin Biotech Co., Ltd, Beijing, China), followed by the appropriate horseradish peroxide-conjugated secondary antibody at room temperature. Finally, the proteins were detected with chemiluminescent substrate.

### ELISA

Wnt3a levels in mouse serum were quantified using a Wnt3a ELISA that was performed according to the manufacturer's instructions Lisu (Shanghai) Biotechnology Co., Ltd, Shanghai, China. All samples were tested in triplicate. Protein levels were calculated as ng/ml of mouse serum.

### Immunohistochemistry

Mice were killed at 3.5 and 5 days post-ABI, and their small intestines were harvested and fixed in neutral formalin. Segments of intestine were collected, dehydrated and embedded in paraffin. Sections that were 3 *μ*m thick were dewaxed and treated with citrate buffer. After antigen retrieval, the sections were treated with hydrogen peroxide for 15 min and then blocked with serum for 1 h. The sections were then incubated with primary antibody, that is, anti-Lgr5, anti-Ki67, anti-lysozyme, anti-CD31, anti-*α*-smooth muscle actin or anti-villin (Abcam), overnight at 4 °C. Sections were washed thoroughly with PBS, and then incubated with secondary antibody for 30 min at 37 °C. After washing, antibody staining was visualized using a DAB kit (ZSGB-BIO, Beijing, China). Sections were counterstained with H&E.

### Apoptosis assay

Mice were killed 6 h post-ABI of 14 Gy, and their small intestines were harvested in entirety and fixed in neutral formalin for histology. Three 5 mm segments of small intestine were taken from each mouse, dehydrated and embedded in paraffin. Sections that were 3 *μ*m thick were dewaxed, rehydrated, treated with proteinase K for 5 min in a 37 °C water bath, and then incubated with TUNEL detection liquid for 1 h at 37 °C. After multiple washes, sections were counterstained with DAPI. These slides were then washed and observed under a fluorescence stereomicroscope.

### Statistical analysis

Data are presented as mean±S.D. One-way ANOVA was used to identify and evaluate differences between groups. If the F distribution was significant, LSD or Tamhane's T2 was used to specify differences between groups. *P*<0.05 was considered statistically significant. The SPSS software package (SPSS Inc., Chicago, IL, USA) was used for the statistical tests.

## Abbreviations

ISCs: intestinal stem cells
MSCs: mesenchymal stem cells
CBCs: crypt base columnar cells
RIII: radiation-induced intestinal injury
ABI: abdominal irradiation
TBI: total body irradiation
PBS: phosphate-buffered saline
TUNEL: terminal deoxynucleotidyl transferase dUTP nick end labeling
TA cells: transit amplifying cells
